# hnRNP A1 Regulates Alternative Splicing of Tau Exon 10 by Targeting 3′ Splice Sites

**DOI:** 10.3390/cells9040936

**Published:** 2020-04-10

**Authors:** Yongchao Liu, Donggun Kim, Namjeong Choi, Jagyeong Oh, Jiyeon Ha, Jianhua Zhou, Xuexiu Zheng, Haihong Shen

**Affiliations:** 1 School of Life Sciences, Gwangju Institute of Science and Technology, Gwangju 500-712, Korea; yongchao@gist.ac.kr (Y.L.); donggunkim@gist.ac.kr (D.K.); njchoi@gist.ac.kr (N.C.); jgoh@gist.ac.kr (J.O.); hajiyn@gist.ac.kr (J.H.); 2 JiangSu Key Laboratory of Neuroregeneration, Nantong University, Nantong 226019, China; jianhua55@msn.com

**Keywords:** alternative splicing, exon 10, Tau, hnRNP A1

## Abstract

The ratio control of 4R-Tau/3R-Tau by alternative splicing of Tau exon 10 is important for maintaining brain functions. In this study, we show that hnRNP A1 knockdown induces inclusion of endogenous Tau exon 10, conversely, overexpression of hnRNP A1 promotes exon 10 skipping of Tau. In addition, hnRNP A1 inhibits splicing of intron 9, but not intron 10. Furthermore, hnRNP A1 directly interacts with the 3′ splice site of exon 10 to regulate its functions in alternative splicing. Finally, gene ontology analysis demonstrates that hnRNP A1-induced splicing and gene expression targets a subset of genes with neuronal function.

## 1. Introduction

Pre-mRNA splicing is a posttranscriptional process in which introns are removed from pre-mRNA and remaining exons are ligated to produce mRNA [1]. Splicing occurs by a large RNA-protein complex called spliceosome that contains U1, U2, U4, U5 and U6 small nuclear ribonucleoproteins (snRNPs) and other proteins [1,2,3]. During splicing, U1 snRNPs recognize 5′ splice sites, U2 snRNPs recognize branch point sequences and U4/U5/U6 snRNPs form a catalytically active splicesome [1,4]. Pre-mRNA contains mainly four splicing signals that are 5′ splice site, 3′ splice site, branch point and polypyrimidine tract (PPT) [1,5,6,7]. Pre-mRNA splicing occurs by two consecutive transesterification reactions: First, the hydroxyl group of adenosine in branch point attacks 5′ end of intron to generate 5′ exon and intron-3′ exon RNA intermediate with a 2′–5′ phosphordiester bond; second, 3′ hydroxyl of the 5′ exon attacks 3′ splice site to result in ligation of the two exons and release of the introns in a lariat form [5,8]. Alternative splicing is a highly regulated process in eukaryotes by which a single gene may generate multiple proteins by differential inclusion of alternative exons in the mature mRNA sequences. RNA sequencing (RNA-seq) analysis in tissues has demonstrated that more than 90% of multiexon genes undergo alternative splicing to generate different mRNA isoforms [1,9,10].

**Figure 1:**
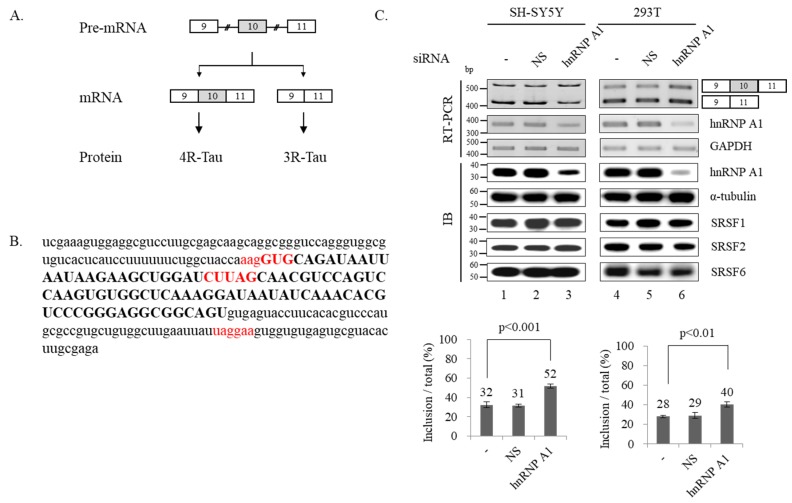
Reduced expression of hnRNP A1 results in increased alternative exon 10 inclusion of Tau pre-mRNA. (**A**) Schematic of Tau pre-mRNA splicing showing two mRNA isoforms produced by alternative splicing of Tau pre-mRNA. (**B**) RNA sequences of exon 10 and its surrounding 80 nt introns are shown. Exon is shown with upper case, introns are shown with lower case. Three potential hnRNP A1 binding sequences are highlighted with red. (**C**) (Upper) RT-PCR analysis of Tau exon 10 splicing in hnRNP A1 knockdown, untreated or non-silencing shRNA treated SH-SY5Y and 293T cells are shown. GAPDH is monitored as a loading control. Immunoblotting of hnRNP A1, α-tubulin, SRSF1, SRSF2 and SRSF6 are shown. (Lower) Bar charts of the exon 10 splicing are shown. *P*-values are shown.

Alternative splicing results in a diversity in neuronal transcriptome and function, conversely, defects in splicing cause neurologic diseases [11,12]. Tau protein is encoded by the microtubule associated protein Tau (*MAPT*) gene and can aggregate to form neurofibrillary tangles that are usually shown in Alzheimer’s disease (AD) and other tauopathies [13,14,15]. There are growing evidences that pre-fibrillar soluble aggregates of Tau or Tau oligomers are primary pathological species that are responsible for the neuronal death [16,17,18,19]. Exon 10 of Tau encodes the second microtubule-binding repeat. Inclusion of exon 10 results in four microtubule-binding repeats (4R-Tau) protein, while exon 10 skipping results in three microtubule-binding repeats (3R-Tau) protein (Figure 1A). In the normal human brain, the ratio of 4R-Tau/3R-Tau is well balanced; however, the ratio is shifted to have excess 4R than 3R in tauopathies and AD [20,21,22,23,24]. Thus, the control of the ratio of 4R-Tau/3R-Tau by alternative splicing of Tau exon 10 is important for maintaining brain functions. Alternative splicing of Tau exon 10 has been shown to be regulated by SRSF1, SRSF2, SRSF3, U2AF, SRSF6, RBM4 and TDP-43 proteins [24,25,26,27,28,29,30,31].

hnRNP A1 is a member of heterogenous nuclear ribonuclear proteins (hnRNPs) [32]. hnRNP A1 contains an *N*-terminal domain with two closely related RRM domains, and a highly flexible glycine-rich (Gly rich) *C*-terminal region with an RGG box RNA binding domain [32,33]. hnRNP A1 is associated with spliceosome, leading to the catalytic excision of the introns and joining of the exons [34,35]. hnRNP A1 forms a complex with U2AF to make the spliceosome select functional 3′ splice sites but not cryptic 3′ splice sites [36]. Roles of hnRNP A1 in alternative splicing were shown in many cases including SMN2, HIV-1 *tat, c- src* exon N1 and Fas [37,38,39,40]. hnRNP A1 has been shown to play key roles in human diseases including genetic deficiencies, cancer development, metastasis, neurodegeneration and replication of viral pathogens [41,42,43,44]. hnRNP A1 expression in the brain is highly reduced in Alzheimer disease patients as well as its mice model [43]. hnRNP A1 knockout mice demonstrated severe muscle developmental defects [45].

In this study, we showed that hnRNP A1 knockdown induces inclusion of endogenous Tau exon 10, conversely, overexpression of hnRNP A1 promotes exon 10 skipping of Tau. We show that hnRNP A1 stimulates exon 10 exclusion without a large part of intron 9. In addition, hnRNP A1 inhibit splicing of intron 9 but not intron 10. Furthermore, hnRNP A1 directly interacts with 3′ splice site of exon 10 to regulate its functions in alternative splicing. Finally, gene ontology analysis demonstrated that hnRNP A1-induced splicing and gene expression targets a subset of genes with neuronal function.

## 2. Materials and Methods

### 2.1. Cell Culture

SH-SY5Y and HEK293T cells were cultured in Dulbecco’s Modified Eagle’s Medium (DMEM) (HyClone, Marlborough, MA, USA) supplemented with 10% fetal bovine serum (FBS) (HyClone), 2 mM Glutamine, 100 U/mL penicillin and 100 μg/mL streptomycin at 37 °C in 5% CO_2_ incubator.

### 2.2. Plasmid Transfection

Cells were seeded 24 h prior to transfection. 0.4 μg plasmid DNAs were mixed with 0.8 μg polyethyleneimide (PEI) reagent in 100 μL DMEM and then incubated at room temperature for 20 min followed by adding to culture plate. Total RNAs were extracted after 48 h.

### 2.3. shRNA Virus Production and Infection

1 μg hnRNPA1 shRNA plasmid was mixed with 0.4 μg of PSPAX2 and PMD2G helper plasmids and then transfected into SH-SY5Y and 293T cell using PEI reagent. Following 24 h incubation, supernatant was harvested by centrifuging at 5000 rpm for 3 min at 4 °C. 300 μL supernatant was mixed with 10 μg/mL polybrene to infect cells for 72 h.

### 2.4. RNA Extraction and RT-PCR

Total RNA was extracted using RiboEX regent (GeneAll, Seoul, Korea) according to instructions from the manufacture. Reverse transcription was performed using oligo-dT_18_ primer and ImProm-II^™^ reverse transcriptase (Promega, Madison, WI, USA) to synthesize first-strand cDNA followed by PCR reaction. In the PCR reaction, a primer pair of E8F/E11R was used to detect alternative splicing of endogenous Tau exon 10, a primer set of pcDNAF/E11R was used to detect exon 10 splicing in Tau minigene, the primer sets pcDNAF/E10R and E10F/pcDNAR were used to detect splicing of intron 9 and 10, respectively. The primer sets A1F/A1R and GAPDHF/GAPDHR were used to detect mRNA expression of HnRNPA1 and GAPDH. The primer sequences are listed in Table S1.

### 2.5. Plasmid Constructions

Tau2, Tau2-1, Tau2-2 and Tau2M were constructed by inserting genomic sequences of Tau into pcDNA3.1^(+)^ plasmid using EcoRI and Xho I (Takara, Tokyo, Japan) by the following primer pairs: E9F(E)/E11R(X), E9F(E)/E9-10R(X), E10-11F(E)/E11R(X) and MutF/MutR. All primer sequences used for constructions are listed in Table S1.

### 2.6. RNA Pull-Down Assay

5′ end biotin-labeled RNA oligos were covalently linked to streptavidin agarose by incubating at 4 °C for 1 h in buffer D (20 mM Tris-Cl pH 7.5, 150 mM KCl, 0.2 mM EDTA, 10% glycerol, 0.5 mM DTT, 0.5 mM PMSF). Following washing with buffer D once, RNA-linked beads were incubated with HeLa cell lysate at 4 °C for 4 h, and then followed by five times washing with buffer D. Beads were loaded onto 12% SDS-PAGE gel and analyzed with immunoblotting assay using anti-hnRNP A1 antibody (Santa Cruz, Dallas, TX, USA; sc-32301). RNA oligo sequences are shown in Table S1.

### 2.7. Immunoblotting Assay

SH-SY5Y and HEK293T cells were lysed in the lysis buffer (0.1% triton X-100, 50 mM Tris-Cl, pH 7.5, 150 mM NaCl, 5 mM EDTA, 1 mM beta-mercaptoethanol) for 30 min at 4 °C, followed by treatment with 5x SDS loading dye and separation using 12% SDS-PAGE gel. After transferring to nitrocellulose membrane, hnRNP A1, SRSF1, SRSF2, SRSF6 and α-tubulin proteins were detected using anti-hnRNP A1 (Santa Cruz; sc-32301), anti-SRSF1 (Santa Cruz; sc-33652), anti-SRSF2 (Millipore, Burlington, VT, USA; 04-1550), anti-SRSF6 (Millipore; MABE152) and anti-α-tubulin (abcam, Cambridge, UK; ab18251) antibodies.

### 2.8. Gene Ontology Analysis and Statistical Analysis

Gene ontology analysis was performed using DAVID Bioinformatics Resources 6.8 (https://david.ncifcrf.gov/) [46]. RT-PCR and immunoblotting experiments were triplicated. Data were presented as mean ± SD (standard deviation of the mean). We performed student’s t-test to obtain the statistical difference between two groups.

## 3. Results

### 3.1. Reduced Expression of hnRNP A1 Results in Increased Alternative Exon 10 Inclusion of Tau Pre-mRNA

To ask the possibility that hnRNP A1 regulates Tau exon 10 splicing, we applied an RNA binding sequence database of RNA binding proteins in humans, SpliceAid (http://www.introni.it/splicing.html) tool, to identify the potential hnRNP A1 binding sequence on Tau exon 10 and around introns. As shown in Figure 1B, we were able to find three potential hnRNP A1 binding motifs located at the 3′ splice site, exon 10 and intron 10. We first tested the possibility that hnRNP A1 regulates alternative splicing of Tau exon 10 with lentivirus-mediated hnRNP A1-targeting shRNA treatment in SH-SY5Y cells, with human neuroblastoma cells, or as a control, non-silencing shRNA treatment followed by RT-PCR of Tau exon 10 alternative splicing. As expected, the shRNAs targeting hnRNP A1 were able to greatly reduce RNA and proteins levels of these hnRNP A1 (Figure 1C; lanes 3), but not GAPDH RNAs and α-tubulin proteins, whereas non-silencing shRNA did not (Figure 1C; lane 2). Notably, hnRNP A1 knockdown (KD) resulted in the significantly increased expression of exon 10 included isoform than untreated or non-silencing shRNA treated cells independently (~21%). We considered a possibility that hnRNP A1 indirectly modulate Tau exon 10 splicing through affecting the expression of other Tau exon 10 regulatory proteins SRSF1, SRSF2 and SRSF6 [25,28,29]. To this end, we tested the expression of these proteins with immunoblotting analysis after hnRNP A1 KD. As shown in Figure 1C, expression levels of SRSF1 SRSF2 and SRSF6 were not altered by hnRNP A1 knockdown (Figure 1C; lane 3). Thus, hnRNP A1 directly regulates alternative splicing of Tau exon 10. We next wondered whether hnRNP A1 also regulates Tau alternative splicing in another cell line. As shown in Figure 1C (lane 6), reduced expression of hnRNP A1 by shRNA in 293T cells also promoted exon 10 inclusion (~11%). As expected, hnRNP A1 did not alter expression of SR proteins including SRSF1, SRSF2 and SRSF6. Thus, the effects of hnRNP A1 + on Tau exon 10 splicing are not limited to one cell line.

### 3.2. hnRNP A1 Promotes Alternative Exon 10 Skipping of Tau Pre-mRNA

**Figure 2:**
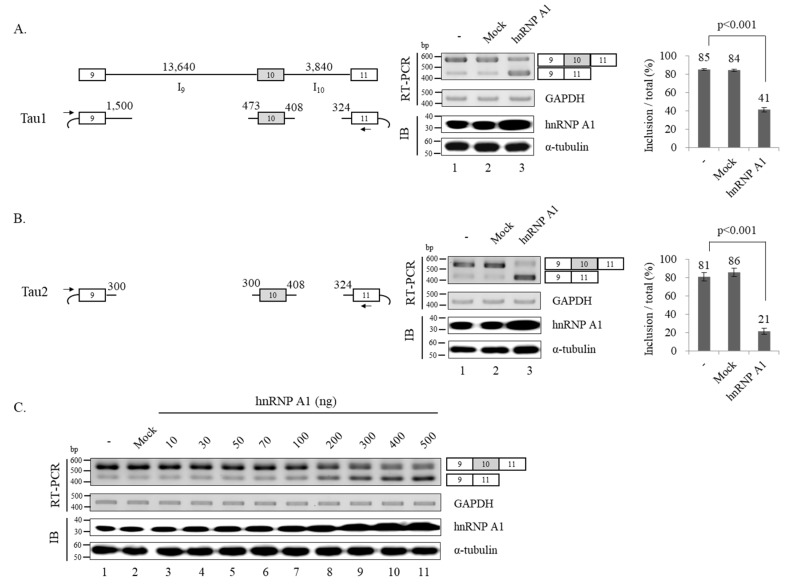
hnRNP A1 promotes alternative exon 10 skipping of Tau pre-mRNA. (**A**) (Left) Schematic of Tau1 minigene showing exons with boxes, introns with lines. Vector sequences are shown with arc. The primers used in RT-PCR analysis are shown with arrows. The length of deleted and remained intron are shown. (Middle) RT-PCR monitoring relative exon 10 skipped isoforms within Tau1 minigene in hnRNP A1 or control pcDNA (3.1) expressed cells. Immunoblotting analysis with anti-hnRNP A1 and α-tubulin are shown. (Right) Bar chart of RT-PCR of Tau1 is shown. (**B**) (Left) Schematic of Tau2 minigene with the length of remained intron 9 and 10. Primers for RT-PCR are shown with arrows. (Middle) RT-PCR analysis of relative exon 10 skipped isoforms within Tau2 minigene in hnRNP A1 or control pcDNA (3.1) expressed cells. (Right) Bar chart of RT-PCR of Tau2 splicing is shown. (**C**) RT-PCR monitoring of alternative splicing of exon 10 in cells transfected with different concentration hnRNP A1. The hnRNP A1 concentrations are shown on the top of lanes. GAPDH is used as a loading control.

As knockdown of hnRNP A1 led to increased level of exon 10 inclusion, we expected that increased expression of this proteins would have opposite effects. However, we could not observe the effects on the endogenous Tau exon 10 splicing. We suspect that it occurred because the cells already have a large amount of endogenous hnRNP A1. Thus, we decided to use Tau minigene system to determine the basis by which hnRNP A1 regulate alternative splicing of Tau exon 10. Tau minigene should contain genomic sequences of exon 9–11 to represent alternative splicing of exon 10. Because intron 9 is 13,640 nucleotides (nt) long and intron 10 is 3,840 nt long, it would be difficult to apply the whole genomic sequences directly to a minigene without any deletions. Thus, we produced a Tau minigene (Tau1) in which only the 5′ end 1,500 nt and 3′ end 473 nt RNA of intron 9 remained, while other parts were deleted; in addition, only the 5′ end 408 nt and 3′ end 324 nt RNA of intron 10 remained whereas other regions were deleted (Figure 2A; left). RT-PCR of Tau1 minigene splicing showed both exon 10 included and skipped isoforms were produced (Figure 2A; middle, lane 1). Notably, overexpressed hnRNP A1 resulted in increased expression of exon 10 skipped isoform and decreased expression of exon 10 included isoform compared to control plasmids (~43%) (Figure 2A; middle, lanes 2 and 3), which are the opposite of the KD effects. We further produced a shorter Tau2 minigene, which included a short (300 nt) 3′ end and 5′ end (300 nt) of intron 9 compared to Tau1 (Figure 2B; left). Figure 2B (middle) shows that hnRNP A1 significantly promoted exon 10 skipped isoform in Tau2 (~65%) (Figure 2B; lane 3). Thus, hnRNP A1 could support exon 10 skipping without the deleted intron 9 RNAs within Tau1 minigene. Because hnRNP A1 promoted exon skipping more efficiently in Tau2 (~65%) than Tau1 (~43%), the deleted RNA might play an important role as an hnRNP A1 repressor.

We next tested the specificity of hnRNP A1 function through transfection of hnRNP A1 plasmids with different concentrations. As shown in Figure 2C, increased amount of hnRNP A1 vector DNA resulted in the increased exon 10 skipped isoform, suggesting the specificity of hnRNP A1 function. Thus, the promoting activity of hnRNP A1 on exon 10 skipping is specific to the proteins. 

### 3.3. hnRNP A1 Inhibits Splicing of Intron 9 but Not Intron 10

**Figure 3:**
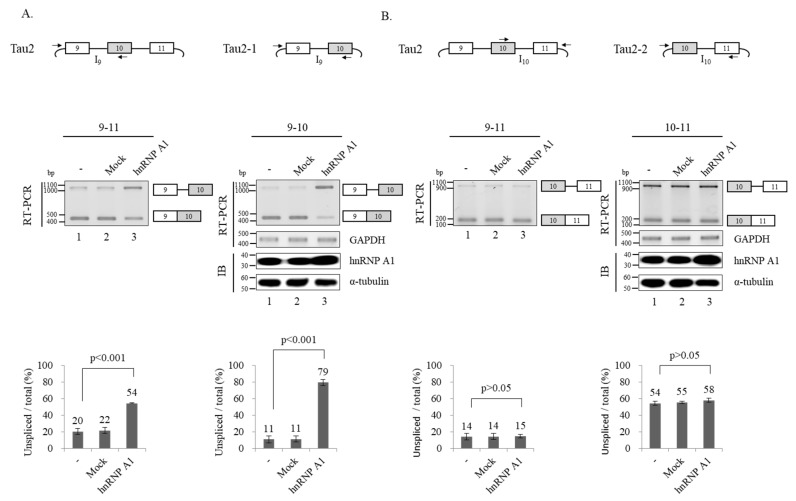
hnRNP A1 inhibits splicing of intron 9 but not intron 10. (**A**) (Upper panel) Schematic of Tau2 and Tau2–1 minigenes are shown. Primers used for RT-PCR are shown with arrows. (Middle panel) RT-PCR monitoring intron 9 splicing in Tau2 and Tau2–1 minigenes in hnRNP A1 or pcDNA transfected cells. Immunoblotting analysis of hnRNP A1 and α-tubulin in Tau-2–1 minigene splicing are shown. Immunoblotting in Tau2 minigene is shown in Figure 2B. (Lower panel) Bar charts of RT-PCR results are shown. (**B**) (Upper panel) Schematics of Tau2 and Tau2–2 minigenes are shown. Primers used for RT-PCR are shown with arrows. (Middle panel) RT-PCR monitoring intron 10 splicing in Tau2 and Tau2-1 minigenes in hnRNP A1 or pcDNA transfected cells. Immunoblotting analysis of hnRNP A1 and α-tubulin in Tau-2–2 minigene splicing are shown. Immunoblotting in Tau2 minigene is shown in Figure 2B. (Lower panel). Bar charts of RT-PCR results are shown.

We next analyzed splicing of two flanking introns of exon 10 to investigate the basis of hnRNP A1-mediated control of exon 10 splicing. To determine the intron 9 splicing, we applied two approaches. First, we performed RT-PCR using Tau2 minigene with a primer set that basepair with a vector sequence located at upstream of exon 9 and exon 10 sequence of Tau2 minigene with the RNAs from Figure 2B (Figure 3A; left). As shown in Figure 3A, hnRNP A1 expression led to detection of intron 9 unspliced isoform (lower), suggesting hnRNP A1 suppressed intron 9 splicing in Tau2 minigene (~32%) (Figure 3A; lane 3). Second, we produced a minigene that harbors exon 9–10 (Tau2–1) (Figure 3A; right) instead of exon 9–11 in Tau2 minigene. Consistent with Tau2 minigene results, hnRNP A1 also inhibited intron 9 splicing in this minigene (~68%) (Figure 3A; lane 2, right). Thus, we conclude that hnRNP A1 inhibits intron 9 splicing of Tau.

Next, we investigated hnRNP A1 effects on intron 10 of Tau. The Tau2 minigene was also used in the study but with a different primer set that basepair with exon 10 sequence or a vector sequence downstream of exon 11 (Figure 3B; left). RT-PCR results using the RNAs from Figure 2B with the different primer set, we showed that hnRNP A1 did not lead to a change of intron 10 splicing compared to the pcDNA vector control (Figure 2B; left lanes 2 and 3). To determine the intron 10 splicing, we produced the Tau2–2 minigene that harbors exon 10–11 (Figure 3B; left). As shown in Figure 3B, hnRNP A1 did not result in the change of the intron 10 splicing. Therefore, hnRNP A1 did not affect intron 10 splicing. Collectively, hnRNP A1 suppressed intron 9 but not intron 10 splicing. 

### 3.4. hnRNP A1 Directly Targets 3′ Splice Site of Alternative Exon 10 to Modulate Alternative Splicing of Exon 10

**Figure 4:**
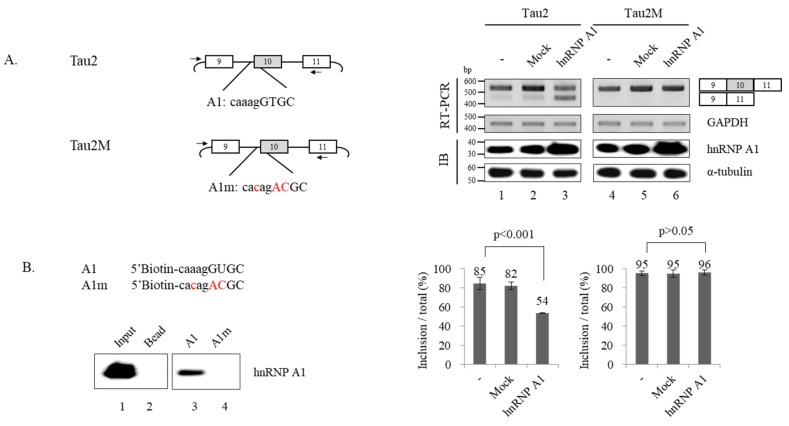
hnRNP A1 directly targets 3′ splice site of alternative exon 10 to modulate alternative splicing of exon 10. (**A**) (Left) Schematic of Tau2 and Tau2M minigene with potential hnRNP A1 binding sequence (A1) and mutated sequences (A1m). The exon sequences are shown with upper case while the intron sequences are shown with lower cases. (Right) RT-PCR monitoring of alternative exon 10 splicing within Tau2 and Tau2M minigenes in hnRNP A1 or control pcDNA expressed cells. Bar charts of RT-PCR are shown. (**B**) (Upper) 5′ biotin labeled RNA oligo sequences of A1 and A1m. (Lower) RNA immunoprecipitation assay monitoring binding of hnRNP A1 to A1 or A1m.

It has been previously reported that hnRNP A1 directly targets RNA to regulate alternative splicing. We considered the possibility that hnRNP A1 regulates alternative splicing of Tau by binding to RNA; thus, we sought to find target sequences of hnRNP A1. Among the three potential hnRNP A1 binding sequences predicted using SpliceAid (Figure 1B), we thought the one located within 3′ splice site of exon 10 might function as the hnRNP A1 target, as it could directly interfere with splice site selection. To test this possibility, we produced a mutant minigene (Tau2M) in which the potential binding sequence of hnRNP A1 (A1: caaagGTGC) was mutated (A1m: cacagACGC) but did not affect 3′ splice site sequence (Figure 4A; left). If this sequence is the functional target of hnRNP A1, mutation would not be able to reduce the expression of exon 10 increased isoform if the promoting effects of hnRNP A1 on exon 10 skipping is considered. In addition, Tau2M minigene would be defective of hnRNP A1 function. Figure 4A shows that Tau2M minigene expressed comparable amounts of exon 10 included isoform as Tau2 minigene (Figure 4A; right, lane 4). Notably, by contrast to Tau2 minigene, hnRNP A1 did not lead to exon 10 skipping of Tau2M minigene (Figure 4A; lane 6). Thus, hnRNP A1 functionally targets the sequence to regulate exon 10 splicing of Tau. We next performed RNA immune precipitation assay using 5′ biotin-labeled A1 RNA oligos to test whether hnRNP A1 interacts with this RNA sequence. We found that hnRNP A1 bound to A1 oligoes (Figure 4B; lane 3) as shown with anti-hnRNP A1 antibody (Figure 4B; lane 3). Conversely, mutated A1m RNA oligoes were not able to interact with hnRNP A1 as expected (Figure 4B; lane 4). Thus, hnRNP A1 interacts with A1 RNA but not mutant A1m oligo. Taken together, we conclude that hnRNP A1 directly targets the RNA sequence within 3′ splice site of exon 10 to regulate exon 10 splicing.

### 3.5. hnRNP A1 Knockdown Affect: Alternative Splicing or Expression of a Subset of Genes with Neuronal Function

**Figure 5:**
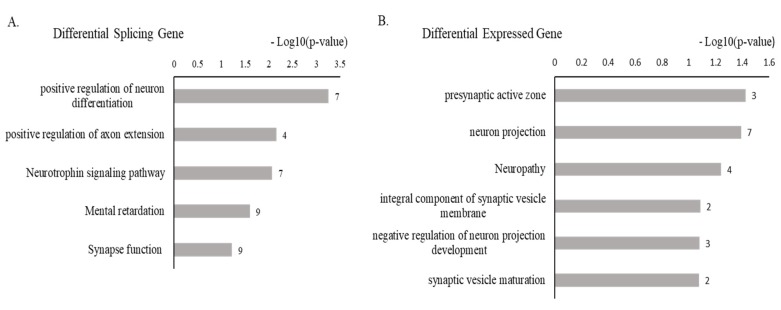
hnRNP A1 knockdown affects alternative splicing or expression of a subsets of genes with neuronal function. (**A**) Gene ontology analysis of differently spliced genes by hnRNP A1. (**B**) Gene ontology analysis of differently expressed genes by hnRNP A1.

The observation that hnRNP A1 regulates Tau alternative splicing made us consider its neuronal roles on global wide scale. To test this possibility, we used RNA-seq data from database. It has been previously reported that hnRNP A1 globally regulates alternative splicing and gene expression, evidenced by RNA-seq using MCF7 cells in which hnRNP A1 knocked down followed by bioinformatics analysis [47]. To determine the gene functions of these affected splicing or expression, we applied gene ontology analysis for the RNA-seq data, which is available at NCBI Gene Expression Omnibus (GEO) via accession number GSE71013. Notably, of the hnRNP A1 affected splicing events, we found a subset of events whose functions are related to neuronal function, including positive regulation of neuron differentiation (7), positive regulation of axon extension (4), neurophrophin signaling pathway (7), mental retardation (9), and synapse function (9) (Figure 5A). Furthermore, of the hnRNP A1 affected expression of genes, we also found a subset of genes with neuronal function that are presynaptic active zone (3), neuron projection (7), neuropathy (4), integral component of synaptic vesicle membrane (2), negative regulation of neuron projection development (3), and synaptic vesicle maturation (2) (Figure 5B). Therefore, hnRNP A1 potentially regulates neuronal roles.

## 4. Discussion

In the current study, we identified hnRNP A1 as an important regulator for the alternative splicing of Tau exon 10. Reduced expression of hnRNP A1 by shRNA knockdown led to increased exon 10 inclusion, conversely, hnRNP A1 overexpression resulted in increased expression of exon 10 skipped isoform in a Tau minigene. In addition, a large part of intron 9 was not required for hnRNP A1 function. Importantly, hnRNP A1 inhibited splicing of intron 9 but not intron 10. Using mutagenesis and RNA immunoprecipitation assay, we demonstrated that hnRNP A1 directly interacted with 3′ splice site of exon 10 to regulate hnRNP A1 function. Gene ontology analysis of RNA-seq database reveal that hnRNP A1 regulated splicing and expression in a subset of genes involved in neuronal function.

Although Tau pre-mRNA contains multiple potential hnRNP A1 binding sites, we have shown that the functional hnRNP A1 target is the one located at 3′ splice site, indicating hnRNP A1 targets the sequences overlapped or close to splice sites. Similar to this result, previous studies have identified the functional targets of RNA binding proteins close to splice sites [37,48,49,50]. One possibility of these target selection is that the shorter distance from splice sites would facilitate the recruitment of splicing factors on splice sites. Although long-distance effects of hnRNP A1 have not been tested in this study, we do not discount the possibility of RNA-binding proteins with the distant functional target sequences. hnRNP A1 has been demonstrated to form a ternary complex with U2AF heterodimer on 3′ splice site; by contrast, hnRNP A1 displaces U2AF from binding to pyrimidine rich RNAs not associated with 3′ splice site [36]. Our results are consistent with previous reports that hnRNP A1 associates with 3′ splice sites.

RNA-seq has demonstrated that significant dysfunction on RNA splicing occurs in the PS19 tau mouse model and in AD brain [51]. In addition, reduced expression of an RNA binding protein TIA1 normalized some of the impaired disease-related RNA splicing [51]. Based on the analysis of the RNA-seq data from database, we found that hnRNP A1 was relevant to neuronal functions as shown by the genes whose splicing and expression are regulated by hnRNP A1 knock down. Whether hnRNP A1 modulating tauopathy or AD brain still needs to be determined.

## Supplementary Materials

The following are available online at https://www.mdpi.com/2073-4409/9/4/936/s1, Table S1: Primer list.
